# Rapidly Progressive Guillain-Barré Syndrome (GBS) as a Critical Predictor of Early Mechanical Ventilation and Prolonged Hospitalization: An Observational Study at a Tertiary Care Hospital

**DOI:** 10.7759/cureus.97916

**Published:** 2025-11-27

**Authors:** Anzil Maharjan, Medha Devkota, Suresh Bishokarma, Ghanshyam Kharel, Pukar S Pakhrin, Sunita Shrestha

**Affiliations:** 1 Neurology, Upendra Devkota Memorial National Institute of Neurological and Allied Sciences, Kathmandu, NPL; 2 Neurology, Charing Cross Hospital, London, GBR; 3 Neurosurgery, Upendra Devkota Memorial National Institute of Neurological and Allied Sciences, Kathmandu, NPL

**Keywords:** duration of hospital stay, guillain-barrè syndrome, mechanical ventilation, rapidly progressive weakness, severe respiratory failure

## Abstract

Background: Guillain-Barré syndrome (GBS) is an acute immune-mediated polyneuropathy that can progress rapidly, often leading to respiratory failure and prolonged hospitalization. Identifying rapidly progressive GBS is crucial for prognostication and timely intervention.

Objective: This study aims to evaluate the clinical progression, need for mechanical ventilation, and hospital stay duration in patients with GBS.

Methods: This is a retrospective observational study of 51 patients diagnosed with GBS. Demographic profile, antecedent events, variants of GBS, treatment offered, need for mechanical ventilation, and duration of hospital stay were studied.

Results: Fifty-one patients with a diagnosis of GBS were included in the study. The mean age of the patients was 36 years, ranging from five to 68 years. The incidence of GBS was higher among males (74.5%) with a male-to-female ratio of 2.92. The mean time of presentation was 10.41±10.42 days (range 1-45 days). Among these, 27 patients (52.9%) presented within a week of symptom onset, while 24 patients (47.1%) presented after a week. Respiratory involvement was seen among 11 Patients (21.6%), while autonomic involvement was seen among nine patients (17.6%). In nerve conduction, acute motor axonal neuropathy (AMAN) was a common variant (31.4%), followed by acute inflammatory demyelinating polyneuropathy (AIDP) (25.5%). Twenty-eight patients (54.9%) received IVIG. Mechanical ventilation was required by 13.7% patients. However, three patients (5.9%) succumbed. Correlation of duration of stay with respect to time of presentation showed a statistically significant negative correlation, suggesting that early presentation is related to severe neurological symptoms, longer hospital stay, and higher respiratory or autonomic dysfunction.

Conclusion: The most common GBS variant in the demographic sampled was the AMAN variant, followed by AIDP. Patients with severe diseases complicated by severe paralytic illness with respiratory and autonomic involvement presented early and had a longer duration of hospital stay. These patients are at high risk of deterioration and, therefore, need to be appropriately managed with timely diagnosis and treatment.

## Introduction

Guillain-Barré syndrome (GBS) is the most common acute paralytic polyneuropathy worldwide, characterized by rapidly progressive ascending weakness, areflexia, and variable sensory and autonomic involvement [1,2]. The disease comprises several subtypes, including acute inflammatory demyelinating polyneuropathy (AIDP) and axonal variants such as acute motor axonal neuropathy (AMAN) and acute motor-sensory axonal neuropathy (AMSAN), which differ in clinical severity, pathogenesis, and prognosis [3]. Despite effective therapies such as intravenous immunoglobulin (IVIG) and plasma exchange, up to one-third of patients develop respiratory failure requiring mechanical ventilation [4,5]. Regional variations in antecedent infections, clinical presentations, and outcomes highlight the importance of local data to guide early recognition and management [6].

Although multiple studies have identified predictors of respiratory failure in GBS, such as bulbar involvement, autonomic dysfunction, and low Medical Research Council (MRC) sum scores, most have examined the overall GBS population rather than focusing specifically on its rapidly progressive phenotype, which may represent a distinct clinical subgroup with higher morbidity. Furthermore, limited data exist from low- and middle-income settings, where delays in diagnosis and treatment can worsen outcomes. This study aims to evaluate the clinical and electrophysiological factors associated with early mechanical ventilation and prolonged hospitalization among patients with rapidly progressive GBS in a tertiary care hospital in Nepal, addressing an important regional and evidence gap.

## Materials and methods

Study design and setting

This retrospective observational study was conducted at a tertiary care center in Kathmandu, Nepal. Medical records of patients diagnosed with GBS during the study period were systematically reviewed to evaluate clinical features, electrophysiological findings, and outcomes.

Data collection

Data were extracted from hospital medical records, including admission notes, progress sheets, and discharge summaries. A structured database was developed to record patient demographics (age, sex, and comorbidities); clinical presentation (antecedent illness, duration and pattern of weakness, autonomic or bulbar dysfunction, and respiratory involvement); laboratory and electrophysiological findings (cerebrospinal fluid (CSF) analysis, magnetic resonance imaging (MRI) findings, and nerve conduction study results); treatment details (use of intravenous immunoglobulin or plasmapheresis, ICU admission, and need for mechanical ventilation); and clinical outcomes, including hospital stay duration, complications, and mortality.

Diagnostic and electrophysiological assessment

The diagnosis of GBS was established based on clinical presentation, CSF findings, and electrophysiological studies in accordance with Brighton Collaboration criteria. Only patients meeting Brighton level 1 or level 2 diagnostic certainty were included. Nerve conduction studies were performed for all patients, and electrophysiological subtypes were classified according to the revised American Clinical Neurophysiology Society (ACNS) criteria (2020) as AIDP, AMAN, AMSAN, or normal variant.

Exclusion criteria

Patients with alternative diagnoses that could mimic GBS were excluded, including those with transverse myelitis presenting in spinal shock, periodic paralysis, lyme disease, or neuroleptospirosis.

Data analysis

Collected data were entered into a secure database and analyzed using appropriate statistical software. Descriptive statistics, including mean, median, and standard deviation, were calculated for continuous variables, while categorical variables were expressed as frequencies and percentages. Group comparisons were performed using the chi-square test for categorical variables and either the independent samples t-test or Mann-Whitney U test for continuous variables, depending on data distribution. A p-value of <0.05 was considered statistically significant.

Ethical considerations

Ethical approval was obtained from the Institutional Review Board (IRB) at Upendra Devkota Memorial National Institute of Neurological and Allied Sciences (UDM-NINAS). The study was conducted in accordance with ethical standards for retrospective research. All patient data were anonymized to maintain confidentiality. As the study was based on retrospective chart review, informed consent was waived by the IRB.

## Results

Between December 2017 and June 2022, 51 patients were admitted to UDM-NINAS who were subsequently diagnosed with GBS. The mean age of the patients was 36 years; the age distribution, however, ranged from a minimum of five years to a maximum of 68 years. Among them, 32 patients (62.7%) were under 40 years of age, and 19 patients (37.3%) were over 40, showing that the incidence of GBS was higher in younger age groups. The incidence of GBS was also higher among males (74.5%) with a male-to-female ratio of 2.92. The time of presentation ranged from 1 day to 45 days, with a mean of 10.41±10.42 days. Among these, 27 patients (52.9%) presented within a week of symptom onset, while 24 patients (47.1%) presented after a week.

The antecedent event could not be elicited in 19 (37%) of patients; however, a preceding diarrhea was reported by 13 (25%) patients, and an upper respiratory tract infection (URTI) was reported as the preceding illness by nine (17.6 %) patients. The study also included the period of the COVID-19 pandemic in 2020-2021; therefore, five (9.8%) of patients developed symptoms of GBS post-vaccination. On analysis of electrophysiological testing, 80% of patients had a typical ascending paralysis starting from the lower limbs, whereas 15% had initial upper limb involvement that later progressed to the lower limbs. About 4% had initial facial nerve involvement. Electrophysiological studies showed that 16 (31.4%) patients had the AMAN variant of GBS, making it the commonest presentation. This was followed closely by AIDP, which affected 13 (25.5%) of patients (Table 1).

**Table 1:** Distribution of biological, investigation, and treatment factor in GBS patients URTI, upper respiratory tract infection; LL, lower limb; UL, upper limb; AIDP, acute inflammatory demyelinating polyradiculoneuropathy; AMAN, acute motor axonal neuropathy; AMSAN, acute motor-sensory axonal neuropathy; IVIG, intravenous immunoglobulin; GBS: Guillain-Barré syndrome

Variables	Component	Frequency	Percent
Antecedent event	None	19	37.3%
	Diarrhea	13	25.5%
	Fever	5	9.8%
	Post-vaccination	5	9.8%
	URTI	9	17.6%
Onset of symptoms	Bilateral facial palsy	1	2.0%
	LL	41	80.4%
	Right facial palsy	1	2.0%
	UL	8	15.7%
Respiratory involvement	Yes	11	21.6%
	No	40	78.4%
Nerve conduction study	AIDP	13	25.5%
	AMAN	16	31.4%
	AMSAN	8	15.7%
	Mixed	12	23.5%
	Normal	2	3.9%
Autonomic involvement	Yes	9	17.6%
	No	42	82.4%
IVIG received	Yes	28	54.9%
	No	23	45.1%
Mechanical ventilation	Yes	7	13.7%
	No	44	86.3%
Outcome	Survive	48	94.1%
	Death	3	5.9%

Respiratory involvement in patients with GBS was assessed by a single breath count test, 11 (21.6%) patients presented with respiratory symptoms. Out of the patients with respiratory involvement, seven patients required mechanical ventilation, which is 64% of patients who presented with respiratory involvement. Autonomic abnormalities in the form of tachyarrhythmias and high blood pressure were observed in nine (17.6 %) patients (Table 1). IVIG could be offered to 28 (55%). Mortality occurred in three (6%) patients, which were patients presenting with early respiratory involvement (Table 1).

The mean duration of hospital stay in patients with GBS was 13 days, with a range of a minimum of 1 day to a maximum of 79 days of hospital admission. A Pearson correlation analysis was performed to examine the relationship between the time of presentation and the duration of hospital stay. As shown in Table 2, there was a statistically significant negative correlation between the two variables (r=-0.285, p=0.043), suggesting that delayed presentation was associated with a shorter hospital stay with milder neurological symptoms and with minimal respiratory or autonomic involvement.

**Table 2:** Correlation between time of presentation and duration of hospital stay ^*^P<0.05 (statistically significant). Note: The correlation is significant at the 0.05 level (2-tailed).

	Time of presentation (days)	Duration of hospital stay
Pearson correlation (r)	1.000	-0.285
P-value (2-tailed)	-	0.043^*^
N	51	51

The study focused on the timing of patients’ presentation to the hospital. Accordingly, the study population was divided into two groups: those who presented within one week of symptom onset and those who presented after one week. The findings, along with p-values and test statistics, are presented in Table 3. Among the 11 patients with respiratory involvement, 30% presented within the first week. Similarly, 26% of patients with autonomic involvement presented within a week. IVIG was administered to 78% of patients who presented within one week of symptoms. Of the seven patients who required mechanical ventilation, six had presented within the first week.

**Table 3:** Comparison based on time of presentation (within one week vs. after one week) ^*^p<0.05 (statistically significant). ^**^p<0.01 (statistically significant). P-values are based on appropriate chi-square or Fisher's exact test as applicable. NCS, nerve conduction study; IVIG, intravenous immunoglobulin; AIDP, acute inflammatory demyelinating polyradiculoneuropathy; AMAN, acute motor axonal neuropathy; AMSAN, acute motor-sensory axonal neuropathy

Variable	Component	Total (n=51)	Within 1 week (n=27)	After 1 week (n=24)	Chi-square	P-value
Respiratory involvement	Yes	11 (21.6%)	8 (29.6%)	3 (12.5%)	2.27	0.130
Autonomic involvement	Yes	9 (17.6%)	7 (25.9%)	2 (8.3%)	2.68	0.100
NCS	Normal	2 (3.9%)	1 (3.7%)	1 (4.2%)	13.09	0.011^*^
NCS	AIDP	13 (25.5%)	4 (14.8%)	9 (37.5%)		
NCS	AMAN	16 (31.4%)	13 (48.1%)	3 (12.5%)		
NCS	AMSAN	8 (15.7%)	6 (22.2%)	2 (8.3%)		
NCS	Mixed	12 (23.5%)	3 (11.1%)	9 (37.5%)		
IVIG	Yes	28 (54.9%)	21 (77.8%)	7 (29.2%)	11.38	0.001^**^
Mechanical ventilation	Yes	7 (13.7%)	6 (22.2%)	1 (4.2%)	3.34	0.068
Outcome	Survived	48 (94.1%)	25 (92.6%)	23 (95.8%)	0.37	0.545
Outcome	Death	3 (5.9%)	2 (7.4%)	1 (4.2%)		

## Discussion

Our study demonstrates a male predominance (74.5%) among GBS patients, aligning with global data that consistently report higher incidence in males, with a male-to-female ratio of approximately 1.5:1 [7]. The majority of cases occurred in individuals younger than 40 years, which is similar to trends reported in several Asian studies but lower than the age distribution typically observed in Western cohorts, where GBS incidence increases with age [8]. A preceding infectious illness was documented in 63% of our patients, most commonly diarrheal or respiratory infections, comparable to existing literature identifying *Campylobacter jejuni* and other pathogens as common antecedent triggers. Notably, 10% of patients in our cohort developed GBS following COVID-19 vaccination, an observation that requires further investigation but is consistent with sporadic reports of post-vaccination GBS in global surveillance studies.

Electrophysiological analysis revealed that AMAN was the most common variant (31.4%), followed by AIDP (25.5%). This contrasts with Western data, where AIDP predominates, accounting for up to 70% of cases [7]. The predominance of AMAN in our population mirrors findings from studies conducted in China, Japan, and other parts of South and East Asia, suggesting potential geographical, genetic, or infectious factors influencing disease subtype distribution. The high prevalence of antecedent gastrointestinal infection in our cohort further supports the hypothesis that *C. jejuni*-induced immune cross-reactivity contributes to axonal pathology in Asian populations [9].

A significant association was observed between early hospital presentation (within one week of symptom onset) and more severe disease manifestations, including rapidly progressive paralysis, respiratory or autonomic dysfunction, and prolonged hospitalization. These findings align with previous studies demonstrating that aggressive subtypes such as AMAN and AMSAN are more likely to present with early respiratory compromise and require mechanical ventilation [10]. The observed outcomes reinforce the importance of early recognition and intervention, particularly timely initiation of IVIG or plasmapheresis, to prevent deterioration and reduce morbidity [11].

Our results underscore the critical need for routine assessment of respiratory and autonomic function in all suspected GBS cases, as well as for multidisciplinary supportive management, including ICU monitoring where indicated. The regional predominance of axonal variants and the high rate of rapidly progressive disease highlight the necessity for context-specific clinical protocols and resource allocation strategies in tertiary care hospitals in Nepal.

While this study provides valuable insights into the clinical and electrophysiological spectrum of rapidly progressive GBS, it is limited by its retrospective design and the lack of long-term follow-up data. Future prospective studies should aim to assess functional recovery and residual neurological deficits post-discharge, which would provide a more comprehensive understanding of disease burden and outcomes in our population.

## Conclusions

Our study identified AMAN as the most common GBS variant, followed by AIDP, consistent with trends in Northern China. A male predominance and younger age of onset were observed compared to global patterns. Patients with severe paralytic illness, respiratory, and autonomic involvement presented earlier and required prolonged hospitalization, highlighting the need for timely diagnosis and treatment. A statistically significant correlation was found between early presentation and longer hospital stay, emphasizing the severity of rapidly progressive cases. Further research into seasonal variations and environmental triggers could provide deeper insights into GBS epidemiology and improve patient outcomes.
